# The Hypersaline Archaeal Histones HpyA and HstA Are DNA Binding Proteins That Defy Categorization According to Commonly Used Functional Criteria

**DOI:** 10.1128/mbio.03449-22

**Published:** 2023-02-13

**Authors:** Saaz Sakrikar, Rylee K. Hackley, Mar Martinez-Pastor, Cynthia L. Darnell, Angie Vreugdenhil, Amy K. Schmid

**Affiliations:** a Biology Department, Duke University, Durham, North Carolina, USA; b University Program in Genetics and Genomics, Duke University, Durham, North Carolina, USA; c Center for Computational Biology and Genomics, Duke University, Durham, North Carolina, USA; University of California, Berkeley

**Keywords:** archaea, histones, regulation of gene expression

## Abstract

Histone proteins are found across diverse lineages of *Archaea*, many of which package DNA and form chromatin. However, previous research has led to the hypothesis that the histone-like proteins of high-salt-adapted archaea, or halophiles, function differently. The sole histone protein encoded by the model halophilic species Halobacterium salinarum, HpyA, is nonessential and expressed at levels too low to enable genome-wide DNA packaging. Instead, HpyA mediates the transcriptional response to salt stress. Here we compare the features of genome-wide binding of HpyA to those of HstA, the sole histone of another model halophile, Haloferax volcanii. *hstA*, like *hpyA*, is a nonessential gene. To better understand HpyA and HstA functions, protein-DNA binding data (chromatin immunoprecipitation sequencing [ChIP-seq]) of these halophilic histones are compared to publicly available ChIP-seq data from DNA binding proteins across all domains of life, including transcription factors (TFs), nucleoid-associated proteins (NAPs), and histones. These analyses demonstrate that HpyA and HstA bind the genome infrequently in discrete regions, which is similar to TFs but unlike NAPs, which bind a much larger genomic fraction. However, unlike TFs that typically bind in intergenic regions, HpyA and HstA binding sites are located in both coding and intergenic regions. The genome-wide dinucleotide periodicity known to facilitate histone binding was undetectable in the genomes of both species. Instead, TF-like and histone-like binding sequence preferences were detected for HstA and HpyA, respectively. Taken together, these data suggest that halophilic archaeal histones are unlikely to facilitate genome-wide chromatin formation and that their function defies categorization as a TF, NAP, or histone.

## INTRODUCTION

The nearly universal conservation of histone proteins across archaeal lineages suggests that eukaryotic histones originated in the domain of life *Archaea*, the evolutionary progenitors of eukaryotes (1, 2). However, the function of archaeal histones in cellular physiology remains less well understood than those of eukaryotes. Eukaryotic histones wrap and compact the genome into a volume small enough to reside inside a nucleus. The four core histones (H2A, H2B, H3, and H4) form an octamer composed of dimer-dimer interactions, and each histone octamer wraps ~147 bp of DNA in a structure called a nucleosome (3). The histone fold domain, which is well conserved between these four proteins, contains residues essential for histone dimerization and histone-DNA interactions (4). A hallmark of histone-based compaction in eukaryotes is their ubiquitous binding throughout the genome, which has been studied using techniques such as chromatin immunoprecipitation coupled to sequencing (ChIP-seq) (5) and micrococcal nuclease digestion sequencing (MNase-seq) (6). While these histones do not have a defined sequence motif, they tend to favor sequences with a 10-bp A/T dinucleotide periodicity, which is thought to facilitate wrapping of DNA around the nucleosome (7).

Certain histone functions of archaeal histones are conserved with those of eukaryotes. This includes DNA compaction, preference for A/T periodic binding sequences, and essentiality. Evidence from Methanothermus fervidus and Thermococcus kodakarensis experiments (including gel shift, nuclease protection, electron microscopy, and X-ray crystallographic structure) suggests that conservation of key residues enables functional retention of DNA wrapping and nucleosome formation (8–11). The strong correspondence between *in vitro* and *in vivo* nuclease protection patterns in these species suggests that their histones compact DNA by binding frequently to periodic sequences throughout the genome (12). Previously, genome-wide 10-bp A/T dinucleotide periodicity was also detected in genomes across a few related thermophilic species (13). Histones across thermophilic lineages are expressed at high levels necessary for genome-wide binding and compaction functions (14). Deletion of a single histone-coding gene in T. kodakarensis resulted in viable organisms; however, strains deleted of both paralogs were nonviable (15).

However, key differences between eukaryotic and archaeal histones have been recently detected. Despite sharing their primary function (i.e., DNA packaging) with eukaryotic histones, T. kodakarensis histones form extended polymeric structures called hypernucleosomes, with individual histone dimers wrapping DNA in multiples of 30 to 60 bp (8, 9, 16). Another thermophilic species, Methanopyrus kandleri, encodes a sole histone protein heterodimer possessing two histone fold domains thought to represent a primordial evolutionary state prior to the emergence of eukaryotic histones (17). *M. kandleri* crystal structures and DNA-histone gel shift experiments suggest nucleosome formation, but it remains unclear whether this species forms hypernucleosomes (11, 17–19). Computational analysis for the many histone paralogs in two mesophilic species, Methanosphaera stadtmanae and Methanobrevibacter smithii, suggested diversification of the paralogs, with some proteins acting as “capstones” blocking histone dimerization and hypernucleosome formation (20). In contrast, in the methanogen Methanosarcina mazei, the lone histone-coding gene is nonessential (21). Instead, evidence has been presented that an archaeal nonhistone chromatin protein called MC1 is involved in genome compaction (22). Therefore, in order to fully understand which aspects of histone function in archaea are conserved with those of eukaryotes, further characterization of histone function across diverse archaeal lineages is needed.

Our previous work in halophilic archaea suggests further diversification of histone functions. HpyA, the sole histone encoded in the genome of the model species Halobacterium salinarum, may function in specific transcriptional regulation of salt-responsive gene expression rather than global chromatin compaction (23). HpyA is expressed at very low levels throughout the growth curve and in chromatin enrichments (24, 25). HpyA structure predictions suggested that it forms a fused histone heterodimer with a highly acidic surface charge, unlike the basic surface of most known histones (24). The *hpyA* gene is readily deleted; the knockout strain exhibits no growth defect under standard conditions relative to the parent strain but is important for growth in reduced salt (23). Global gene expression is dysregulated during low-salt stress in an Δ*hpyA* strain, and discrete, infrequent peaks of binding enrichment were observed in ChIP-seq data under low-salt conditions. In contrast, other archaeal histones show ubiquitous genome-wide binding (8, 12). Taken together, these data are inconsistent with a role for HpyA in genome-wide DNA compaction as has been observed for other archaeal histones (8) but instead suggest a role in condition-specific transcriptional regulation. However, an HpyA *cis*-regulatory binding motif and binding enrichment in promoter regions were undetectable, and HpyA regulates its target genes in a largely indirect manner. These results suggest further complexities to HpyA function (23). As in *Hbt. salinarum*, the genomes of the hypersaline-adapted order of the *Archaea*, *Halobacteriales*, encode a fused histone heterodimer whose surface acidity and primary amino acid sequence are well conserved throughout the order (24), suggesting that these histones may have evolved novel cellular roles by duplication and divergence (1, 26); however, the functions of histones in other halophilic archaeal species remain unexplored.

Here we extend the knowledge of halophilic histones by studying the genome-wide binding patterns of HstA, the sole histone protein of another model halophile, Haloferax volcanii, and by comparing it to HpyA. Although both *Hbt. salinarum* and *Hfx. volcanii* are members of the same phylogenetic order, the two species are members of different clades of the *Halobacteriales* and are therefore model representatives of halophilic archaeal phylogenetic diversity (27). Both species are extreme halophiles; however, the extracellular salt concentration supporting optimal growth differs (4.2 M NaCl for *Hbt. salinarum* versus 2.5 M NaCl for *Hfx. volcanii*) (28, 29). Given the phylogenetic and physiological divergence of these organisms but sequence conservation between halophilic histones, we compare HpyA and HstA binding patterns to better understand the broader functional conservation of halophilic histones. To gain further insight into halophilic histone function, we compared binding characteristics of HpyA and HstA more broadly with those of known DNA binding proteins across domains of life, including bacterial nucleoid-associated proteins (NAPs); archaeal, eukaryotic, and bacterial transcription factors (TFs); and archaeal and eukaryotic histones. Specifically, we compared characteristics of sequence specificity, binding location (intergenic versus coding), binding frequency, start site occupancy, and binding peak size and shape. Together, these data suggest that HpyA and HstA possess a medley of conserved and unique DNA binding functional features.

## RESULTS

### Haloferax volcanii histone HstA is not essential for viability but is important for maintaining wild-type growth rate.

**FIG 1:**
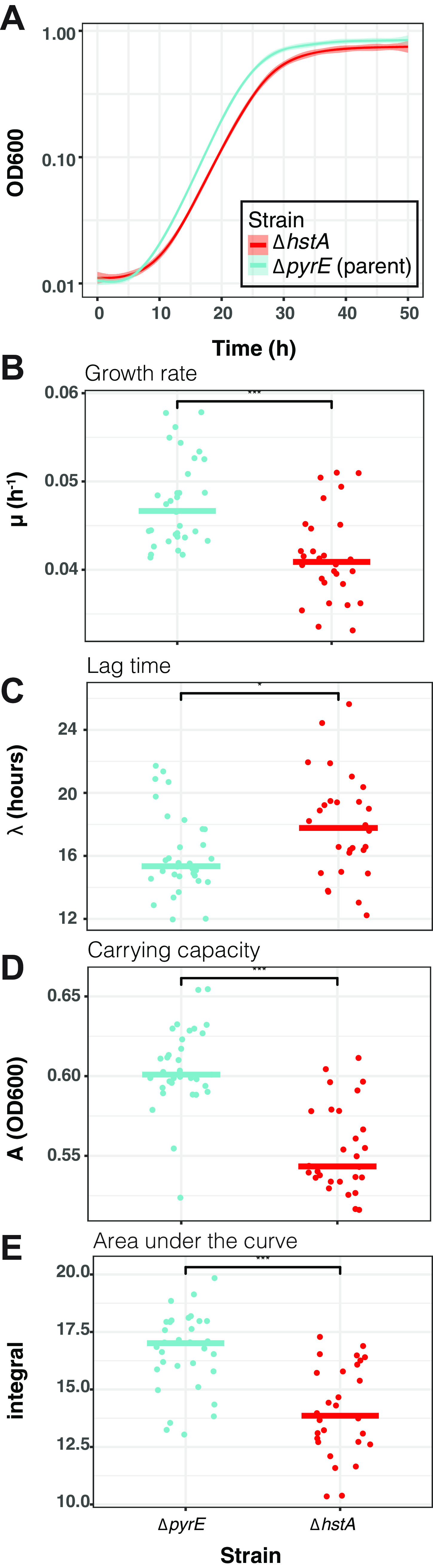
HstA is important for growth under optimal conditions. (A) Growth of strains (Δ*pyrE*, blue; Δ*hstA*, red; 9 biological replicates with 2 to 3 technical replicates each) measured as optical density (OD_600_). The heavy lines represent the smoothed conditional mean growth curves; the shaded area surrounding each curve represents the error of the mean. Colors shown in the key are consistent throughout all panels. (B) Logistic growth rate (μ, per hour) of parent versus mutant strain. (C) Lag time (λ, hours). (D) Carrying capacity in stationary phase (OD_600_). (E) Area under the log-transformed curve (integral). In each graph, each dot represents one technical replicate growth curve. Horizontal lines represent the median of the distribution of points for each strain. Brackets with asterisks show the results of a Welch two-sample *t* test: ***, *P* < 0.001; *, *P* < 0.05.

HstA (HVO_0520) is the sole histone protein encoded in the genome of the model halophile *Hfx. volcanii*. As we observed in our previous study (24), HstA shares 65% sequence identity with HpyA histone-like protein of *Hbt. salinarum* and retains residues conserved across histones of nearly 80 sequenced halophile genomes. HpyA was shown to be nonessential (i.e., able to be deleted) with no change in growth relative to the parent strain (23, 24). We used a genetic approach to compare the role of *Hfx. volcanii* HstA in growth to that of HpyA. We observed that *hstA* was readily deleted from *Hfx. volcanii* (details in Materials and Methods), suggesting that it is nonessential, similar to *hpyA.* However, unlike the Δ*hpyA* deletion strain of *Hbt. salinarum*, the Δ*hstA* strain exhibited a significant growth defect compared to the parent strain under optimal conditions (rich medium at 42°C) (Fig. 1A; see also Table S1 at https://doi.org/10.6084/m9.figshare.19391648), including lower growth rate (Fig. 1B, Welch two-sample *t* test *P* < 0.001), longer lag time (Fig. 1C, *P* < 0.05), lower carrying capacity in stationary phase (Fig. 1D, *P* < 0.001), and smaller area under the curve (Fig. 1E, 84% of parent strain, *P* < 1.5 × 10^−6^). Growth was also tested under a variety of stress conditions (sodium and magnesium stress, oxidative stress with peroxide, and alternate nutrient conditions). The growth of the Δ*hstA* strain under these conditions was 84% to 91% of the growth of the parent control strain, which was at or above the level for optimal conditions (see Fig. S1 at https://doi.org/10.6084/m9.figshare.19391648). These data indicate that the Δ*hstA* growth defect under standard conditions is not further compounded by stress, suggesting that *hstA* is dispensable for growth under the conditions tested. The growth defect under standard conditions is significantly complemented by the in *trans* expression of *hstA* driven by the native promoter (see Fig. S2 at https://doi.org/10.6084/m9.figshare.19391648). Complementation is observed when *hstA* is expressed alone or translationally fused in frame to the hemagglutinin epitope tag, indicating that the growth defect is attributable to the deletion of *hstA* and not due to polar effects on surrounding genes. In addition, these data indicate that the C-terminal hemagglutinin (HA) tag does not interfere with HstA function, allowing ChIP-seq with the tagged strain to be carried out. Whole-genome resequencing verified the absence of any secondary site mutations in this strain (Table S1) and the complete absence of any wild-type *hstA* copies from the genome (halophiles are highly polyploid [30], necessitating such validation). Taken together, these results establish that *hstA* can be deleted but is important for growth under standard conditions. We conclude that HstA resembles *Hbt. salinarum* HpyA in that both can be deleted but differs in that HstA is important for growth under standard conditions.

### The halophilic histones HpyA and HstA bind throughout the genome in a pattern intermediate between transcription factors and nucleoid-associated proteins.

**FIG 2:**
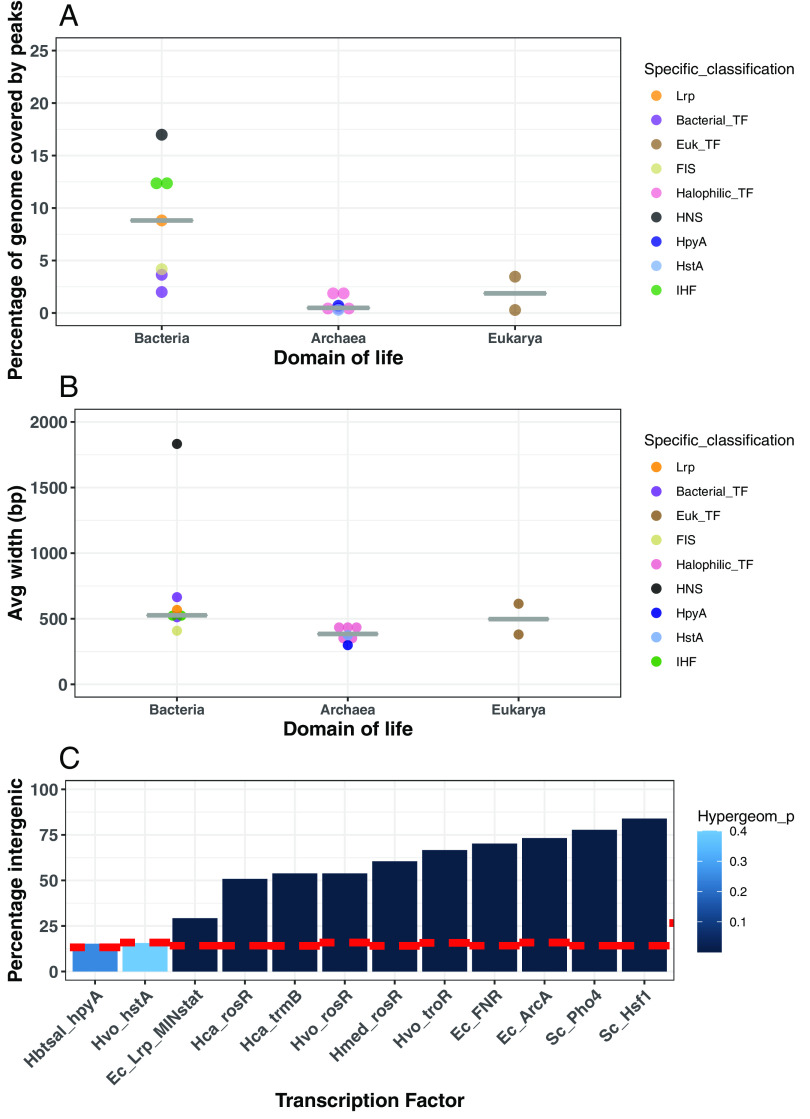
Genomic features of HpyA and HstA binding sites according to ChIP-seq data. (A) Percentage of genome covered by all ChIP-seq peaks of a given DNA binding protein across domains of life, arranged into columns by domain of life. Dots are colored by protein type as shown in the key. (B) Average width of all ChIP-seq peaks for a given DNA binding protein, arranged into columns by domain of life. (C) Bar graph of ChIP-seq peaks for HpyA, HstA, and transcription factors TrmB, RosR, TroR, FNR, ArcA, Pho4, Hsf1, and Lrp (“Lrp_MINstat” indicates minimal medium stationary-phase conditions). Species names are abbreviated as follows: Hbtsal, *Hbt. salinarum;* Hvo, *Hfx. volcanii*; Hca, *Hca. hispanica;* Hmed, *Hfx. mediterranei;* Ec, Escherichia coli*;* Sc, S. cerevisiae. The height of each bar represents the percentage of peaks located in intergenic regions. The dashed red line indicates the percentage of each genome that is noncoding. The intensity of color of the bars represents hypergeometric test *P* values of significance for enrichment within promoter regions (see key for color scale).

To classify the binding patterns of halophilic histones as a proxy for function, we compared ChIP-seq binding patterns of HpyA (23) and HstA to those of TFs, NAPs, and eukaryotic histones. HstA data were newly acquired in the current study under optimal conditions in exponential phase, whereas data for the other proteins were drawn from the literature (5, 31–40). Across the *Hfx. volcanii* genome, 32 reproducible binding sites were observed for HstA, which represents <1% of the genome bound (Fig. 2A) (see also Tables S2 and S3 at https://doi.org/10.6084/m9.figshare.19391648). A comparable number of peaks covering a small fraction of the genome were also observed in published data for *Hbt. salinarum* HpyA (<1%), TFs from halophilic archaea (0.4 to 2.2%), bacteria (2 to 3.7%), and eukaryotes (0.3 to 3.4%) (Fig. 2A; see Table S3 at the URL mentioned above). Consistent with this infrequent and punctuated binding, the average width of HpyA and HstA binding footprints (299 bp and 374 bp, respectively) is comparable to those of TFs across domains of life (299 to 665 bp; Fig. 2B). In contrast, the average peak width and percentage of the genome covered by binding sites of the various bacterial NAPs are generally higher and more variable (Fig. 2A and B). Mean peak widths range from 408 bp (FIS) to 1,832 bp (H-NS) (Fig. 2A). On average, NAP binding sites cover a larger fraction of the genome (average 11%) than halophilic histone and TF binding peaks, particularly in the case of H-NS (Fig. 2B and see Table S3 at the URL mentioned above). Lrp exhibits a mix of characteristics: its average peak width (567 bp) is typical for bacterial TFs, but an intermediate percentage of genome was covered (8.8%) (Fig. 2 and see Table S3 at the URL mentioned above).

Neither HpyA nor HstA shows enrichment for genic or intergenic sequences (*P* value > 0.4; see Table S4 at https://doi.org/10.6084/m9.figshare.19391648). However, unlike HpyA, which regulates ion uptake (23), the genes near HstA binding peaks were not enriched for a particular function according to archaeal Clusters of Orthologous Genes (arCOG) categories (41). In contrast, TF binding sites were, as expected, significantly overrepresented in intergenic regions relative to the genomic backgrounds of the corresponding species, which are 84 to 87% coding (hypergeometric test; *P* value < 1 × 10^−3^) (Fig. 2C and see Table S4 at the URL mentioned above). The proportion of TF peaks binding to intergenic regions varied from 51% for Haloarcula hispanica TrmB to 84% for Saccharomyces cerevisiae Hsf1 (Fig. 2C). Hence, while halophilic histones appear to bind without preference for genic or intergenic regions, TF binding favors intergenic regions. For Lrp, although its binding is significantly enriched in intergenic regions, <30% of peaks are located in intergenic regions.

**FIG 3:**
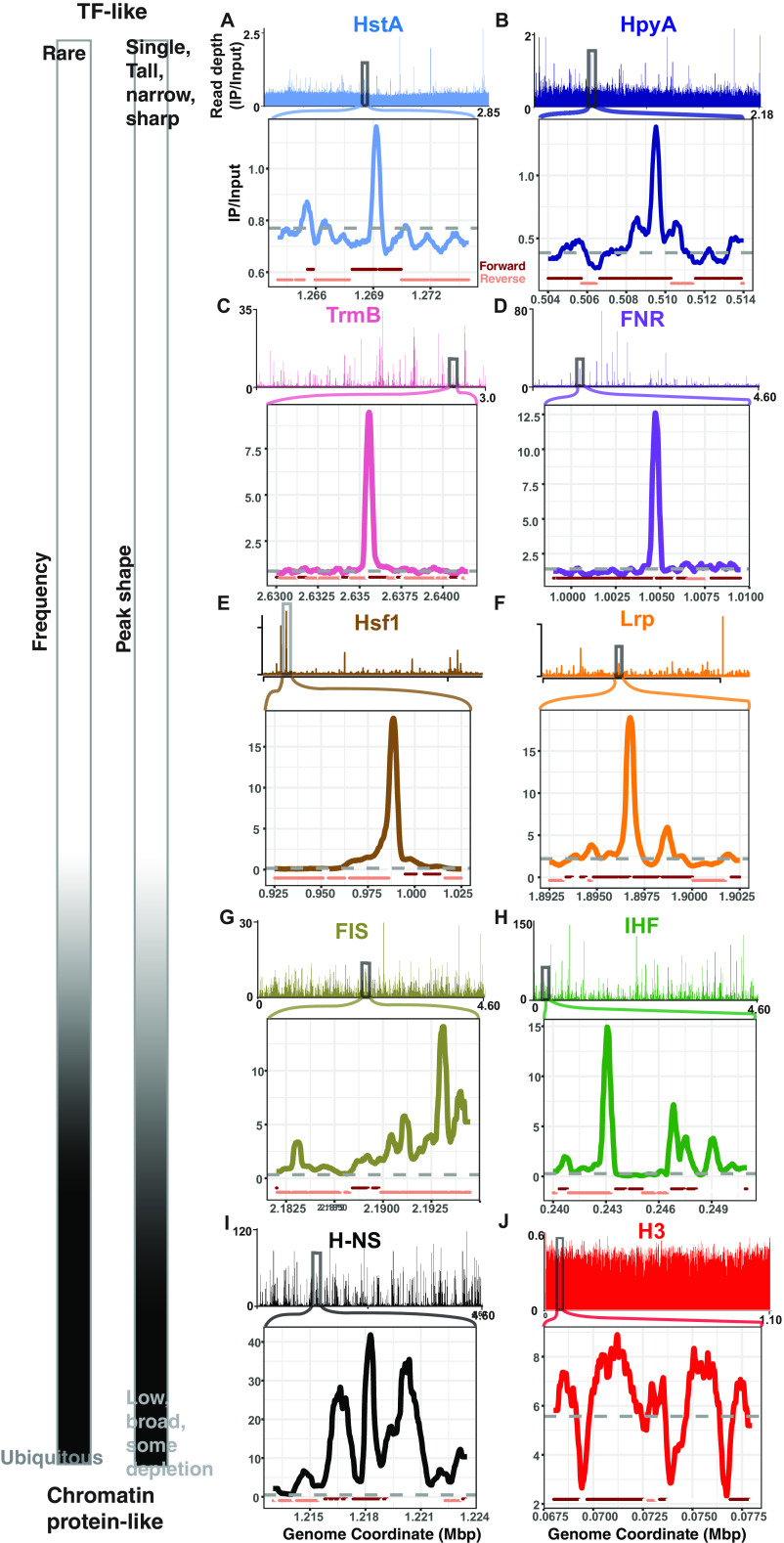
ChIP-seq binding signal for HpyA and HstA compared with TFs, NAPs, and eukaryotic histone. In each panel, chromosome-wide binding patterns (measured as read depth of IP/input) are shown above, and zoomed-in regions of representative peaks are shown below. All archaeal and bacterial genome views depict the main chromosome of each species. (A) *Hfx. volcanii* HstA (light blue, NCBI accession NC_013967.1, zoom-in peak center located at 1.27 Mb). (B) *Hbt. salinarum* HpyA (dark blue, NC_002607.1, peak center at 0.51 Mb). (C) Halophilic TF Haloarcula hispanica TrmB (pink, NC_015948.1, peak center at 2.64 Mb). (D) Bacterial TF E. coli FNR (purple, NC_000913.3, peak center at 1.01 Mb). (E) Yeast Saccharomyces cerevisiae Hsf1 (brown, chromosome XVI, NC_01148.4, peak center at 0.988 Mbp). (F) E. coli Lrp (orange, NC_000913.3, peak center at 1.897 Mbp). (G) FIS (olive). (H) E. coli IHF (green). (I) E. coli H-NS (black, peak center at 1.22 Mb). (J) Yeast histone H3 (red, chromosome VII, NC_001139.9). For the TFs and H-NS, known to directly regulate target genes (31, 37, 78), peaks with a known functional role were chosen. For each genome-wide view and zoom-in, the *x* axis represents chromosomal coordinates in megabase pairs (Mbp), and the *y* axis represents the read depth ratio of IP to input control (i.e., binding enrichment). Gray dashed lines in the zoom-ins represent a baseline calculated from the average genome-wide IP/input signal; dark red and tan lines below each zoom-in plot represent genomic context (forward and reverse strand genes, respectively). The scale at left indicates the classification of each DNA binding protein pattern based on features of frequency and peak shape.

Close visual inspection of the genome-wide binding patterns and individual peaks reflected these quantitative observations. Discrete, narrow regions of binding enrichment at relatively few locations in the genome were observed for halophilic histones, TFs, and Lrp (Fig. 3A to F and see Fig. S3A to C at https://doi.org/10.6084/m9.figshare.19391648). Both narrow and broad peaks were observed for NAPs (Fig. 3G to I and see Fig. S3D at the URL mentioned above), consistent with their multiple molecular roles in the cell (42). In contrast, binding peaks were not observed for yeast histones in a genome-wide view (see Fig. S3E at the URL mentioned above). However, local genomic regions exhibited broad, flat areas of enrichment punctuated by depletion at gene promoters (Fig. 3J).

Taken together, these observations suggest that HpyA and HstA DNA binding peak width and frequency resemble those of site-specific TFs that function in condition-dependent transcriptional regulation via promoter binding (43). However, halophilic histone genomic binding locations are more like those of Lrp or NAPs, which are agnostic for binding genic versus intergenic sequences.

### Halophilic archaeal histone protein occupancy curves surrounding start sites are unique relative to canonical histone and TF signals.

**FIG 4:**
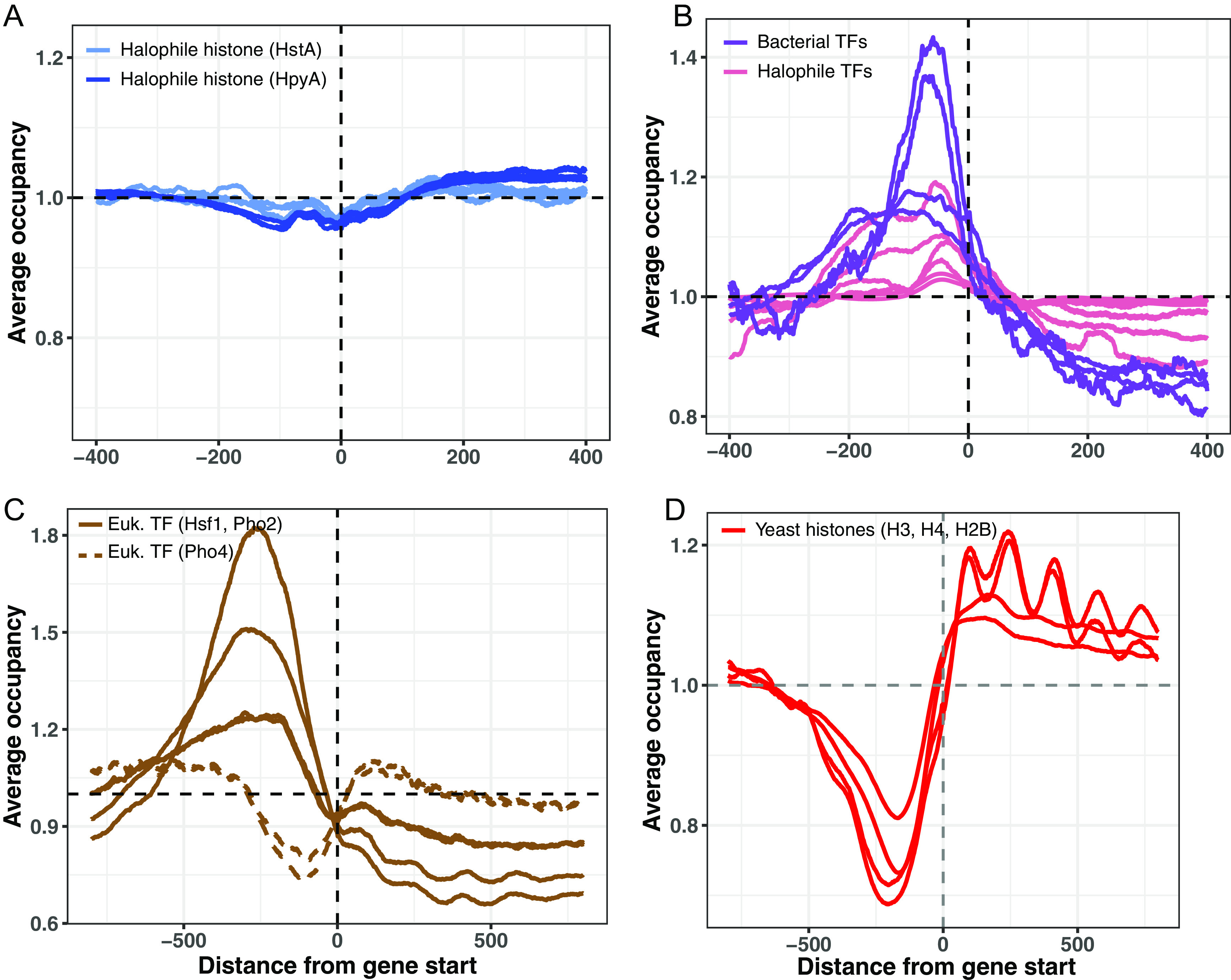
Binding occupancy at start sites of selected DNA binding proteins. (A) Average binding occupancy across all genes for HstA (light blue, 1 representative replicate from each condition tested) and HpyA (dark blue, 3 replicates, where each line is one replicate). In each panel, the *x* axis represents distance from start site (base pairs), and the *y* axis represents average occupancy, as measured by read depth in genomic positions around the start site, normalized to average depth across the genome. (B) Bacterial TFs E. coli ArcA and FlhD (purple) and archaeal TFs *Hca. hispanica* TrmB, *Hfx. volcanii* TroR, *Hfx. mediterranei* RosR (pink, 2 replicates each). (C) Eukaryotic TFs Hsf1 and Pho2 (solid lines) and Pho4 (dashed lines). (D) Yeast histones (red; each line represents a biological replicate experiment).

To further investigate how HpyA and HstA bind DNA, the average occupancies (i.e., normalized read depths) at open reading frame (ORF) start sites were compared across DNA binding proteins (see Materials and Methods). While HpyA and HstA occupancy was not enriched at any particular location within 500 bp of gene start sites (Fig. 4A), occupancy of TFs of halophilic archaea, bacteria, and eukaryotes was enriched 50 to 250 bp upstream (Fig. 4B and C). A majority of these TFs also show a slight but variable depletion of occupancy within the gene body (Fig. 4B and C, solid lines), except the eukaryotic TF Pho4 (Fig. 4C, dashed lines). Previous studies demonstrated that Pho4 access to most promoter regions is inhibited by the presence of nucleosomes but that Pho4 binds strongly at a subset of accessible promoters (32). Bacterial Lrp occupancy resembled that of TFs, while NAP occupancy was variable, including upstream enrichment and/or depletion (see Fig. S4A and B at https://doi.org/10.6084/m9.figshare.19391648). As expected, yeast histone occupancy was depleted in the promoter region (~160 to 200 bp from the start site) but enriched at regular intervals indicative of nucleosome binding (~100 to 150 bp apart) (Fig. 4D). Heatmap representations showing occupancy data for individual start sites across the genome highlight the striking differences between the eukaryotic histone occupancy profiles (H3, see Fig. S5A and B at https://doi.org/10.6084/m9.figshare.19391648) and the halophilic archaeal histone profiles (HpyA; see Fig. S5C and D at the URL mentioned above). Taken together, this occupancy analysis reveals a unique binding pattern for halophilic histones compared to all other DNA binding proteins considered here.

### HpyA and HstA are predicted to bind DNA using unique sequence determinants.

**FIG 5:**
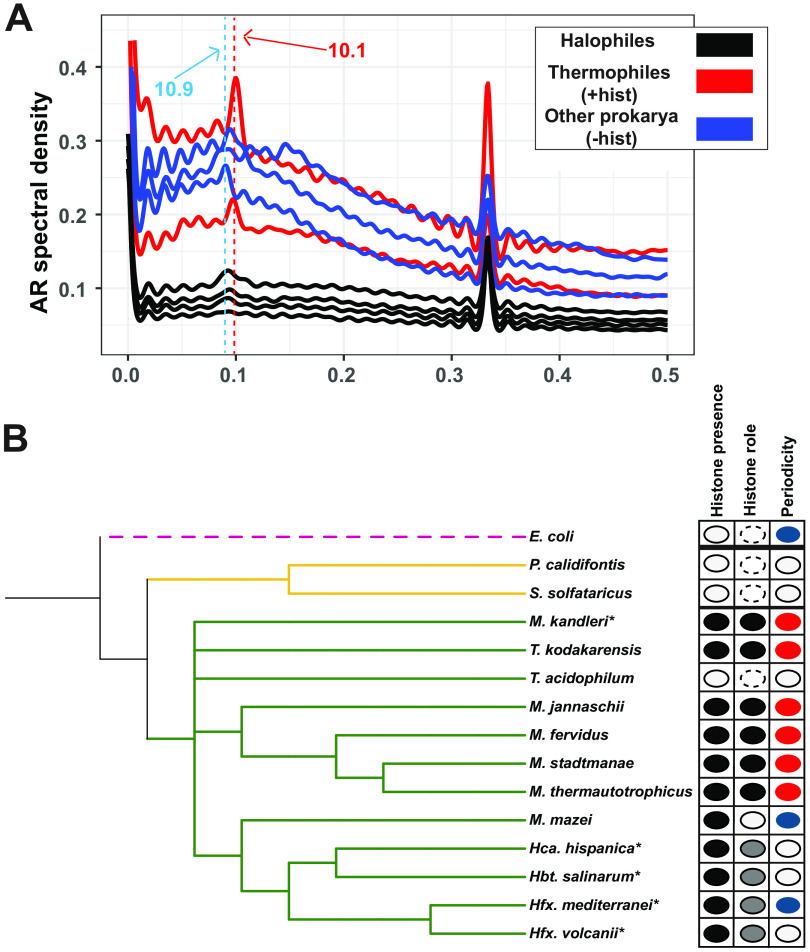
AA/TT/TA dinucleotide periodicity shows histone-linked pattern. (A) Autoregression (AR) spectra indicating genome-wide dinucleotide periodicity of thermophilic archaeal species with well-characterized histones (Methanothermus fervidus, Thermococcus kodakarensis; red lines), halophilic archaea that encode histones (*Hbt. salinarum*, *Hfx. volcanii*, *Hfx. mediterranei*, *Hca. hispanica*; black traces), and other prokaryotic species (blue traces) that lack histones (E. coli, Sulfolobus solfataricus) or with nonhistone chromatin (*M. mazei*). Dashed red line indicates ~10.1-bp periodicity present in histone-utilizing species (red traces); dashed blue line represents ~10.9-bp periodicity (i.e., from supercoiling) detected in some non-histone-utilizing species (blue traces). Note that the strong peak at 0.33 bp^−1^ (3 bp) seen in all these spectra is linked to codon usage; it is present in all species and is not linked to histone binding (75). (B) Phylogenetic tree of selected archaeal species (with the bacterium E. coli as the outgroup). The first column shows detectable (black) or undetected (white) histone-encoding genes. The second column documents experimental characterization of the function of the encoded histone based on previous publications: compaction (black), noncompaction (white), noncanonical histone function (gray), or not detected (dashed line). The third column indicates the genome-wide AA/TT/TA dinucleotide periodicity: ~10 bp (red), ~11 bp (blue), or no detectable periodicity (white). Species marked with an asterisk (*) indicate high GC content. Only the *M. kandleri* genome carries the GC periodicity signal (see Fig. S6 at https://doi.org/10.6084/m9.figshare.19391648 for GC periodicity graphs).

To provide additional information regarding halophile histone functions, we next investigated sequence determinants of binding. Because a genome-wide 10-bp dinucleotide periodicity signal (GPS) can be indicative of histone packaging (7, 13), power spectrum analysis was conducted to detect the GPS of AA/TT/TA dinucleotides in the genome sequences of diverse organisms (Materials and Methods; also see Table S5 at https://doi.org/10.6084/m9.figshare.19391648) (13). The genomes of thermophilic archaeal species that encode histones with characterized roles in chromatin formation exhibit a sharp peak in their respective spectral density curves at 10 to 10.3 bp (Fig. 5A and see Fig. S6A at https://doi.org/10.6084/m9.figshare.19391648) (8, 9). In contrast, in archaeal and bacterial genomes lacking histones, periodicity of 10.7 to 11 bp (indicative of negative supercoiling) was detected for some species, whereas periodicity was undetectable for others (Fig. 5A, blue traces) (44). Although the genomes of the four model halophilic species considered here each encode a histone, AA/TT/TA periodicity was not detected (Fig. 5A, black traces, and Fig. 5B, species marked with asterisks). In these high-G+C% genomes, 10-bp GC periodicity was also not detected compared with a control (Fig. S6B). These trends hold across a wide array of archaeal species: 10-bp A/T GPS is detected in genomes of species known to use histones to form chromatin, whereas periodicity was not detected for those lacking histones (Fig. 5B) (12, 20, 45).

**FIG 6:**
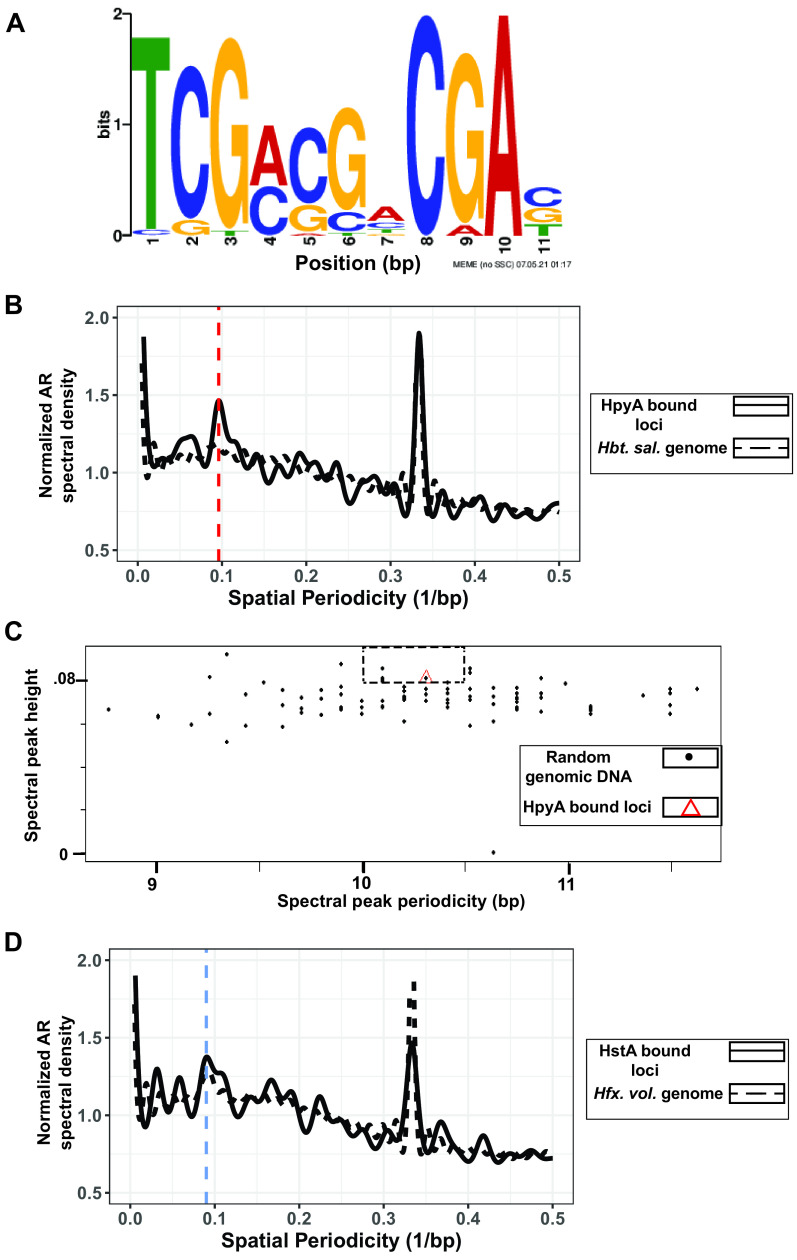
Sequence specificity of HpyA and HstA binding. (A) Motif logo of *cis*-regulatory sequence detected in HstA-bound sites. Bit scores are shown on the *y* axis and base pair positions on the *x* axis. Motif logo generated by the MEME suite output (46). (B) The 10.4-bp periodicity is present in HpyA-bound loci (solid black line) but absent in the *Hbt. salinarum* genome (dashed black line). The vertical red dashed line indicates 10 bp. (C) Comparing randomly chosen regions of the genome (black dots) with the periodicity of the HpyA-bound loci (red triangle). The dashed rectangle includes those randomly chosen sequences that show stronger periodicity than HpyA at relevant levels (10 to 10.5 bp). (D) The ~11-bp frequency of HstA-bound loci (black solid line) matches the periodicity of the entire genome of *Hfx. volcanii* (black dashed line). The blue dashed line indicates 11 bp.

Given that GPS was undetectable in halophile genomes, we searched *de novo* for specific *cis*-regulatory sequence motifs associated with HpyA and HstA binding using programs such as MEME (46), DNA secondary structures, and overrepresented k-mers (Materials and Methods; see also Supplementary File S1 at https://doi.org/10.6084/m9.figshare.19391648). In the case of HstA, a palindromic sequence in 31 of the 32 ChIP-seq peaks was detected (Fig. 6A, E value, 1.4 × 10^−14^). The TCGNSSNCGA (where S is G or C) motif was robust to correction for background di- and trinucleotide frequencies. Genome pattern scanning analysis using FIMO (part of the MEME suite) detected this motif at 11,630 locations genome-wide, suggesting that HstA may bind additional sites under alternate conditions. In contrast, exhaustive *de novo* computational searches using multiple methods were unable to detect a sequence-specific binding motif for *Hbt. salinarum* HpyA (details in Supplementary File S1 at the URL mentioned above). Instead, we asked whether HpyA binding regions specifically exhibit a 10-bp GPS. Surprisingly, a periodicity of 10.4 bp was indeed detected in HpyA-bound regions although the GPS was not detected at a genome-wide level (Fig. 6B). Three of 100 randomly chosen sequences across the genome (equal to the length of the HpyA-bound regions) exhibited greater spectral peak height (indicating stronger periodicity) in the 10- to 10.5-bp range (Fig. 6C). This suggests that HpyA may bind additional sites in the genome and/or under alternative growth conditions that have not yet been investigated. In contrast, HstA target loci exhibited 11-bp periodicity but not ~10-bp periodicity (Fig. 6D). Together, these results suggest that the GPS can be used as a sequence-based predictor of genomic dependence on histones for nucleosome and chromatin formation and that unique sequence determinants facilitate HpyA and HstA DNA binding.

## DISCUSSION

This study investigated the conserved and unique DNA binding patterns of histone-like proteins across two related species of halophiles. The sole histone coding gene of two halophilic species is nonessential for growth. However, unlike HpyA of *Hbt. salinarum*, HstA of *Hfx. volcanii* is important for growth under optimum conditions (Fig. 1). Comparison of ChIP-seq data for TFs, NAPs, and histones across domains of life revealed that HpyA and HstA DNA binding patterns represent a pastiche of conserved and unique features. The genome-wide binding of HstA and HpyA are similar with respect to number, width, and shape of binding peaks; percentage of genome covered; and lack of preference for genomic features. However, HpyA and HstA differ in terms of their sequence preferences.

Considering the evidence presented here, we conclude that HpyA and HstA functions diverged from those of other archaeal and eukaryotic histones. First, they are not essential for viability. Genes encoding known chromatin proteins are usually essential (15, 47, 48), if not individually, then combinatorially. For example, Thermococcus kodakarensis encodes two different histone proteins. Although each single deletion strain is viable, deletion of both genes is lethal (15). Second, HpyA in *Hbt. salinarum* and HstA in *Hfx. volcanii* are expressed at low levels comparable to those of TFs and, correspondingly, bind in discrete peaks covering <1% of the genome (14, 24, 49) (Fig. 2 and 3). In contrast, chromatin proteins are typically highly expressed and bind at sites covering at least 10% of the genome (Fig. 3) (6, 14, 36, 37, 50). More broadly across the archaeal phylogenetic spectrum, histone expression level is strongly associated with chromatinization of the genome (14). Third, the absence of a 10-bp periodicity signal in *Hfx. volcanii* suggests that the nuclease-protected regions detected in previous reports were likely bound by a protein other than histone (see Fig. S6C and D at https://doi.org/10.6084/m9.figshare.19391648) (51). In contrast, in archaeal species whose histones are known to function in chromatin organization, both a 10-bp GPS and nuclease protection by nucleosomes are observed (see Fig. S6D at the URL mentioned above) (8, 12). High expression levels, frequent binding, 10-bp genomic periodicity, and nuclease protection are associated with histone-like genomic architectural functions. Therefore, evidence presented here suggests that halophilic histones are unlikely to play a role in facilitating global genome architecture. Other predicted chromatin proteins in *Hfx. volcanii* are expressed at much higher levels than HstA (HVO_1577, HVO_2029) (14, 49) and are therefore alternative candidates for chromatin organization in this species.

Instead, HstA and HpyA exhibit a medley of DNA binding features that suggest their function has diverged from characterized eukaryotic and archaeal histones. Across the genome, halophilic histone binding enrichment peaks are sparse, punctuated, and narrow, which is similar to those observed for TFs whose binding is typically highly enriched at short, specific *cis* sequences (31, 33) (Fig. 2 and 3). In contrast, HpyA and HstA binding peaks differ from NAPs, whose peaks were wide, broad, and frequent, consistent with their roles in DNA architecture and compaction (36, 37, 52–54). For example, H-NS is an NAP investigated here that binds in peaks spanning ~1 kb, consistent with its known biological role in transcription silencing (55) and bridging of supercoils (37, 56). Unlike those of TFs and yeast histones, HpyA and HstA binding occupancy is not enriched at a particular location across start sites (Fig. 4 and see Fig. S4A at https://doi.org/10.6084/m9.figshare.19391648). This pattern is consistent with a lack of preference for binding genic versus intergenic regions (Fig. 3C). Like eukaryotic and other archaeal histones, HpyA favors binding in discrete DNA regions with an ~10-bp dinucleotide periodicity despite the lack of genome-wide periodicity (Fig. 6). In contrast, HstA target loci exhibited 11-bp periodicity but not ~10-bp periodicity (Fig. 6D). HpyA and HstA could therefore play a role in local genome architecture. At HstA binding sites, a semipalindromic *cis*-regulatory consensus sequence motif was detected, more like site-specific TFs (Fig. 6A). Taken together, these data suggest that HpyA and HstA binding patterns resemble those of TFs in some respects and those of NAPs and histones in others.

**FIG 7:**
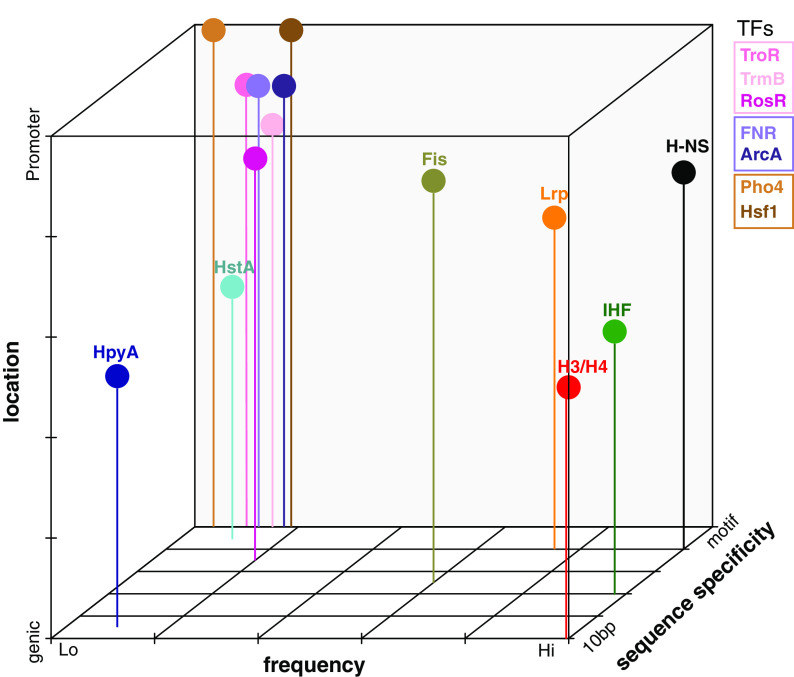
Visual summary of binding characteristics of selected DNA binding proteins investigated in this study. Binding is classified based on sequence specificity, ranging from preference for 10 bp periodicity (10 bp) to strict *cis* sequence motif (motif); frequency as measured by genome-wide coverage and number of peaks, ranging from low (Lo) to high (Hi); and location preference, ranging from coding to promoter preference. Figure is qualitative; tick marks and gridlines are intended for visual clarity.

Our results therefore situate HpyA and HstA in a growing group of DNA binding proteins that defy categorization according to commonly used criteria. Based on our comparative analysis, we observe that TFs tend to group more closely together in terms of their binding location, frequency, and specificity, compared with greater variation in these binding features across NAPs (Fig. 7). Consistent with our findings, Dorman and colleagues have posited that traditional definitions of bacterial DNA binding proteins as “TFs” or “NAPs” are insufficient to capture the true continuum of functional characteristics observed for certain proteins (57). For example, some proteins were defined as NAPs because of their ability to bind genome-wide and alter DNA structure; however, some NAPs can also bind in a highly sequence-specific manner (e.g., IHF). Some TFs like cyclic AMP receptor protein (CRP) exert sequence-specific control of certain loci but bind to hundreds of other sites in the genome (58, 59). Such examples are not restricted to bacteria: newly discovered site-specific archaeal TFs are also likely to bend or loop DNA. Examples include the TetR family TF FadR (60, 61) and archaeal Lrp family proteins (62, 63). Depending on the locus and nutrient conditions, Lrp family proteins can bind with or without sequence specificity (40) and exhibit direct or indirect effects of transcription (39). The DNA binding proteins under investigation in the current study therefore require more flexible functional categorization, and we have provided systematic quantitative criteria for comparing DNA binding proteins across domains of life using ChIP-seq data. It would be interesting in future studies to explore additional criteria such as how binding patterns change dynamically over time and across a variety of growth and/or stress conditions. For example, Lrp binding patterns resemble those of either TFs, NAPs, or both, depending on growth conditions, reflecting their condition-dependent functional roles (39). However, dynamic changes in binding across a time course have not yet been conducted for Lrp. Similarly, it is known that HpyA binds more frequently under low-salt conditions (23); however, it remains unclear how HstA changes its binding profiles under alternate growth conditions.

Given the strong conservation of the single fused histone heterodimer across sequenced halophile genomes (24, 27), we posit that alternative functions are likely for other halophilic histones. However, given the mosaic of functional features observed here for HpyA and HstA, future research is needed to determine how broadly conserved those alternative functions may be across the halophiles. Nevertheless, our results are consistent with the hypothesis that archaeal histone function varies according to habitat through the process of selection under extreme conditions (14).

In summary, we conclude that HpyA and HstA—with their primary sequence homology to archaeal and eukaryotic histones, their role as transcription regulators (23, 24), and hybrid modes of DNA binding—lie within the unclear divide between TFs, histones, and nucleoid-associated proteins.

## MATERIALS AND METHODS

### Strain construction.

The Haloferax volcanii wild-type strain used in this study was DS2 (64). The strains created here used the DS2 derivative Δ*pyrE* strain (strain H26) (65) as the parent strain. The Δ*hstA* (*HVO_0520*) knockout strain AKS198 was created from parent H26 using vectors described by Allers et al. (66) and the pop-in pop-out double crossover counterselection strategy commonly used for *Hfx. volcanii* (66). Briefly, the pAKS145 vector for creating the knockout was generated by isothermal ligation of sequences flanking the *hstA* gene into backbone vector pTA131 at the EcoRV site. Strains, primers, and plasmids used for all strain constructions are noted in Table S6 at https://doi.org/10.6084/m9.figshare.19391648.

AKS214 was the strain used to test in *trans* complementation of the Δ*hstA* deletion growth defect. It contains the pAKS147 plasmid, which was created by inserting *hstA* and 500 bp of its upstream sequence into the pJAM809 backbone at the XbaI and KpnI sites. Two strains were generated for ChIP-seq experiments. AKS217, the negative-control strain, is the Δ*hstA* background carrying the pJAM809 empty vector (67). AKS233 is the Δ*hstA* strain carrying plasmid pAKS180, which was derived from pAKS147 by addition of the hemagglutinin (HA) tag using the New England Biolabs (NEB) Q5 site-directed mutagenesis kit.

*hstA* deletion from the genome and *hstA* or *hstA-HA* presence in *trans* were confirmed with PCR and Sanger sequencing of the flanking regions. Deletion was additionally confirmed with full-genome resequencing. Full-genome resequencing for the parent strain and Δ*hstA* strain was analyzed using the *breseq* (68) analysis tool; results are given in Table S6 at https://doi.org/10.6084/m9.figshare.19391648.

### Media, culturing, and phenotyping.

*Hfx. volcanii* rich medium, yeast peptone Casamino Acids (Hv-YPC), was used for routine growth across experiments as described previously (66). For plasmid maintenance, media were supplemented with novobiocin (0.1 μg/mL). For construction of deletion mutants, media were supplemented with 5-fluoroorotic acid (5-FOA) (300 μg/mL) in selection of the second crossover.

To measure growth rates of the Δ*pyrE* parent strain and Δ*hstA* strains, at least 3 biological replicate individual colonies of H26 and AKS198 were picked from plates freshly streaked from frozen stock and precultured for 70 to 80 h in 5 mL Hv-YPC at 42°C with 225-rpm shaking (referred to as “standard” or “optimum conditions” here). To test growth phenotypes under standard conditions, precultures were diluted to an optical density at 600 nm (OD_600_) of ~0.025 and then cultured in a Bioscreen C (Growth Curves USA) at 42°C with fast shaking at maximum amplitude. Each biological replicate culture was inoculated into at least duplicate and up to quadruplicate wells of the microtiter plate to ensure technical reproducibility in the measurements. OD_600_ was measured by the Bioscreen every 30 min over the growth curve. Further details of stress conditions were tested, and results are given in Fig. S1 at https://doi.org/10.6084/m9.figshare.19391648. The logistic model fit to each resultant growth curve was used to calculate time in lag phase (λ), growth rate (μ), asymptotic carrying capacity in stationary phase (*A*), and the integral (area under the log-transformed growth curve [AUC]). Fitting was performed using the grofit package in the R statistical environment (69). Visualizations and graphing were carried out using the ggplot2 package in R (70). Code for these analyses is available at https://github.com/amyschmid/Halophilic_histone_binding/tree/main/Growth_analysis. Raw growth data for the Δ*hstA* strain and parent strain under optimal conditions are provided in Table S1 at https://doi.org/10.6084/m9.figshare.19391648.

### ChIP-seq experiment.

Haloferax volcanii HstA-HA ChIP-seq was carried out using methods described previously (23). Briefly, three biological replicate cultures of AKS233 (*hstA*-HA) and 1 replicate of AKS217 as a negative control were grown in 50 mL of YPC18% and harvested at 15 to 17 h postinoculation at an optical density of 0.21 to 0.33 (mid-exponential phase). Cultures were cross-linked and immunoprecipitated by means of the HA tag, and DNA was prepared as described previously (71). Strain details are provided in Table S6 at https://doi.org/10.6084/m9.figshare.19391648. As before, the Duke Center for Genomic and Computational Biology carried out library preparation including adapter ligation. The only difference from previous protocol was the use of the Illumina NovaSeq6000 to carry out paired-end sequencing.

### ChIP-seq analysis.

Publicly available ChIP-seq data for the relevant TFs, NAPs, and histones were downloaded from the NCBI Sequence Read Archive using the fastq-dump feature from SRAToolkit 2.9.0 (https://hpc.nih.gov/apps/sratoolkit.html). Details of published data sets used for bacterial and archaeal TFs, histones, and NAPs, including Sequence Read Archive accession numbers and complete citations, are provided in Table S5 at https://doi.org/10.6084/m9.figshare.19391648. Technical criteria for inclusion of ChIP-seq data were as follows: (i) data were freely available through supplemental material and/or online repositories, (ii) they included an input control, and (iii) they included at least 2 reproducible replicate experimental trials. Biological criteria for inclusion of DNA binding proteins were (i) known function in site-specific transcriptional regulation (“TF-like” functions), (b) known function in DNA architecture, or (c) primary amino acid sequencing homology with HpyA and HstA. Briefly, these DNA binding proteins included halophilic archaeal TFs (*Hca. hispanica* RosR and TrmB, *Hfx. volcanii* RosR and TroR, and Haloferax mediterranei RosR [Schmid lab, unpublished data]), bacterial NAPs (IHF, H-NS, FIS), bacterial TFs (FNR, ArcA, FlhD), eukaryotic TFs (Pho2, Pho4, Hsf1), halophilic histones (HstA, HpyA), and eukaryotic histones (H3, H4, H2A, H2B). Fastq files were converted to sorted BAM files and wig files, and per-base read-depth text files were generated as described previously (23).

Sorted BAM files were used to find regions enriched for TF binding (immunoprecipitation [IP] versus input control, i.e., ChIP-seq “peaks”) using the R package MOSAiCS (72). For experiments where more than one replicate from the same conditions was present, multiIntersectBed from the BEDTools package (73) was used to combine peaks across replicates, and only peaks present in the majority of replicates were considered. Peaks detected in yeast mitochondrial DNA were excluded due to inconsistencies in read coverage. Excluded from peak finding in general were FlhD and Pho2 because of spurious peaks in MOSAiCS analysis; yeast histones were also excluded due to a general lack of binding peaks and presence of depletion regions. Peaks detected in the zoom-in graphs (Fig. 2) were chosen based on three criteria: (i) they represent functional binding sites verified by orthogonal data sets, (ii) they are among the strongest peaks in the data set, and (iii) they depict a typical location with multiple peaks (in the case of NAPs). The code used to generate this is provided at https://github.com/amyschmid/Halophilic_histone_binding/tree/main/Peak_calling.

The peak list for HpyA was taken from our previously published work (23). The peak list for HstA was created as described above but with some manual curation (details below). The average width and total area covered by the peaks within these lists were calculated within Microsoft Excel, and total area covered was expressed as a percentage of genome length (see Table S3 at) https://doi.org/10.6084/m9.figshare.19391648.

Peaks were classified as “intergenic” or “coding” based on where in the genome they were located. The center of each peak was found and was determined to be within or outside a coding region (as described by the list of genes in the NCBI gene table for that species). The code used to make this classification and to graph the results is at https://github.com/amyschmid/Halophilic_histone_binding/tree/main/Bindingfeatures.

The results of this classification were used as the basis of a hypergeometric test in R using the phyper function to determine if peaks were overrepresented in intergenic regions (see Table S4 at https://doi.org/10.6084/m9.figshare.19391648).

### Generating *Hfx volcanii* HstA peak list.

MOSAiCS was used to generate peak lists from HstA ChIP-Seq data, and peaks common in at least 2 of 3 replicates were retained to make a joint peak list. This list was then curated manually to remove false positives caused by changes in input control sequencing, transposase and integrase-caused local duplications, and peaks common with the HA tag-alone input control. The final manually curated peak list for HstA is noted in Table S2 at https://doi.org/10.6084/m9.figshare.19391648.

### Transcription start site occupancy analysis.

Per-base read-depth text files were generated as described above for all DNA binding proteins of interest here (see Table S5 at https://doi.org/10.6084/m9.figshare.19391648). These text files were used as inputs for occupancy analysis, alongside genome annotations downloaded from NCBI (details in Table S5 at the URL mentioned above). Code was written that returns a matrix where each row corresponds to a single gene, and the columns represent sequence depth at positions from ±400 bp of that gene’s start site, normalized to the average depth over the whole chromosome. For yeast histones and TFs, this analysis was repeated by changing the boundaries to ±800 bp; we did this to accommodate the larger size of intergenic regions and the known 147-bp length of DNA bound to a single nucleosome. The occupancy graph was generated by taking the average of occupancy across all start sites (rows in the matrix). The code used for this analysis is at https://github.com/amyschmid/Halophilic_histone_binding/tree/main/TSSgraphs.

Note that the start site used here refers to the ORF translation start site instead of the transcription start site (TSS) that is often used for these analyses. Three criteria motivated this choice: (i) ORF start sites are better annotated in most species, including halophilic archaea; (ii) ORF and transcription start sites are encoded within a few base pairs in genomes of halophiles, with >60% of ORFs being leaderless in *Hfx. volcanii* (74); and (iii) using ORF start sites, we were able to reproduce previously seen patterns for yeast TSS (5) (Fig. 4A).

### Dinucleotide periodicity analysis.

FASTA files containing the genome sequence of the relevant species were downloaded from the NCBI website (species and download details in Table S5 at https://doi.org/10.6084/m9.figshare.19391648) and were analyzed using custom R scripts. In brief, dinucleotides (AA/TT/TA) are detected in each genome and binarized: locations with these dinucleotides are marked as 1 and the rest of the genome as 0. Then, the autoregression spectrum spec.ar function in the R stats package (with default parameters) was used to estimate the spectral density of this binary signal, which indicates the periodicity of the selected dinucleotides using an autoregression fit. For facilitating clarity in visualization of autoregression curves, periodicity was normalized by the average signal. The same analysis was carried out for GC dinucleotides (see Fig. S6B at https://doi.org/10.6084/m9.figshare.19391648). For nucleosome enrichment analysis, data regarding the center of the nucleosomes were downloaded from supplementary information of the work of Brogaard et al. (6) (for Saccharomyces cerevisiae), Maruyama et al. (8) (for Thermococcus kodakarensis), and Ammar et al. (52) (for Haloferax volcanii). Sequences of the length of a typical nucleosome (150 bp for eukaryotes and 30 to 60 bp for archaea) were isolated around each center, and the same analysis as described above was carried out. Note that the strong peak at 0.33 bp^−1^ (3 bp) seen in all these spectra is linked to codon usage; it is present in all species and is not linked to histone binding (75). Depending on the AT content of the sequence being examined, some of the spectra have an increasing or decreasing slope resulting from slight deviations in A+T content locally; this too is not linked to histone binding (75). The codes used for these analyses are available at https://github.com/amyschmid/Halophilic_histone_binding/tree/main/Periodicity_genomewide. The phylogenetic tree for Fig. 5B was made using the Integrated Tree of Life (iTOL, https://itol.embl.de).

### Motif search.

Bed files containing peak locations from HstA and HpyA ChIP-seq data were converted to FASTA format using the BEDTools (73) getfasta command. These FASTA files were used as input for various motif and overrepresented sequence determining programs. We used motif detection with MEME (46) and Homer (76), k-mer detection tool KMAC (77), and a DNA secondary structure detection R-package called gquad (https://cran.r-project.org/web/packages/gquad/index.html). Finally, the fasta-get-markov tool of MEME was used to determine background mono-, di-, and trinucleotide frequencies. A more detailed description of the parameters used for each program, and the results of the searches, is provided in Supplementary File S1 at https://doi.org/10.6084/m9.figshare.19391648.

For obtaining periodicity of sequences bound by HpyA or HpyA, the FASTA file containing all the peaks (generated as described above) was merged into a single line. Periodicities of the sequences in this FASTA file were analyzed. A method similar to the procedure used to analyze genome-wide periodicity was used here. The obtained periodicity was compared with randomly chosen sequences from the genome roughly equal in number and length to the width of ChIP-seq peaks (peak widths given in Table S2 at https://doi.org/10.6084/m9.figshare.19391648 and in reference 23). These simulated peak lists were then analyzed using the autoregression spectrum scripts, and results were compared between the 100 simulated sequences and the empirically detected peaks.

### Data availability.

The ChIP-seq data have been deposited in the National Center for Biotechnology Information (NCBI) Gene Expression Omnibus (GEO) under accession number GSE186415. The whole-genome sequencing data for the Δ*hstA* deletion strain have been deposited in the NCBI Sequence Read Archive at accession no. PRJNA773760. All code and input data for analyses presented here are freely available via the GitHub repository https://github.com/amyschmid/Halophilic_histone_binding. All supplemental figures, tables, and documents are available on Figshare at https://doi.org/10.6084/m9.figshare.19391648.
